# Free-Energy-Based Discrete Unified Gas Kinetic Scheme for van der Waals Fluid

**DOI:** 10.3390/e24091202

**Published:** 2022-08-27

**Authors:** Zeren Yang, Sha Liu, Congshan Zhuo, Chengwen Zhong

**Affiliations:** 1 School of Aeronautics, Northwestern Polytechnical University, Xi’an 710072, China; 2 National Key Laboratory of Science and Technology on Aerodynamic Design and Research, Northwestern Polytechnical University, Xi’an 710072, China

**Keywords:** free-energy model, discrete unified gas-kinetic scheme, multiphase flow, flux reconstruction

## Abstract

The multiphase model based on free-energy theory has been experiencing long-term prosperity for its solid foundation and succinct implementation. To identify the main hindrance to developing a free-energy-based discrete unified gas-kinetic scheme (DUGKS), we introduced the classical lattice Boltzmann free-energy model into the DUGKS implemented with different flux reconstruction schemes. It is found that the force imbalance amplified by the reconstruction errors prevents the direct application of the free-energy model to the DUGKS. By coupling the well-balanced free-energy model with the DUGKS, the influences of the amplified force imbalance are entirely removed. Comparative results demonstrated a consistent performance of the well-balanced DUGKS despite the reconstruction schemes utilized. The capability of the DUGKS coupled with the well-balanced free-energy model was quantitatively validated by the coexisting density curves and Laplace’s law. In the quiescent droplet test, the magnitude of spurious currents is reduced to a machine accuracy of $10^{-15}$. Aside from the excellent performance of the well-balanced DUGKS in predicting steady-state multiphase flows, the spinodal decomposition test and the droplet coalescence test revealed its stability problems in dealing with transient flows. Further improvements are required on this point.

## 1. Introduction

Multiphase fluid flow characterized by the concurrent presence of multiple thermodynamic phases is frequently encountered in industrial processes and engineering applications. Insightful understanding of the multiphase flow behavior could facilitate improvements in manufacturing technology and production efficiency. Due to the restriction on measurement technology and the experimental platform, it is particularly challenging to reveal the flow details by experimental methods. Benefiting from the substantial improvements in computing power, numerical simulation technology has been developed into a powerful tool for the study of complicated behaviors arising in multiphase fluid flow. By numerically solving the set of interface capturing and hydrodynamic equations, a multitude of research studies [1,2,3,4] vividly detail the interface dynamics and flow structures from a macroscopic perspective. Essentially, the interfacial phenomenon represents the macroscopic manifestation of the microscopic interactions among fluid molecules [5]. Numerical methods based on realistic microscopic physics could offer in-depth findings regarding multiphase phenomena, but the heavy computational requirement of such methods for industry-scale multiphase problems is far beyond affordable. In recent years, numerical schemes constructed with the mesoscopic theory [6] have been emerging as a compelling methodology for resolving multiphase flow patterns as this bridges the gap between the macroscopic descriptions of multiphase dynamics and microscopic intermolecular interactions and, thus, generates insightful understandings at an affordable cost.

Among various previously proposed mesoscopic approaches [7,8,9], the lattice Boltzmann (LB) method [7] has received particular attention for its concise and intuitive way of representing intermolecular interactions. Generally, the lattice Boltzmann multiphase models developed in the past few decades can be categorized into four classifications: the color-gradient model [10], the phase-field model [11,12], the pseudopotential model [13], and the free-energy model [14]. The phase-field model employs independent sets of distribution functions to separately transfer mass and momentum, which could cause mass non-conservation problems near the interface region [15]. The pseudopotential model and the free-energy model employ a single set of distribution functions to ensure a coherent transport of mass and momentum, which conforms to the physical reality that mass and momentum are simultaneously transferred by the unique molecules. Compared to the pseudopotential model, where interactions are built heuristically, the free-energy model is constructed upon the stationary-action principle, which possesses a firm physical background. Over the last couple of decades, the free-energy lattice Boltzmann method has been successfully applied to numerically tackle a variety of flow issues including the contact line movement [16,17], multicomponent fluids’ flow [18,19], wetting boundaries [20,21], and large-density-ratio fluid flow [22,23]. The primitive free-energy multiphase model proposed by Swift et al. [14] reflects the interaction effects via a modified equilibrium distribution function, whose second-order moment incorporates a nonideal thermodynamic pressure tensor. However, this primitive model suffers from a lack of Galilean invariance due to the superfluous terms recovered in the momentum equation. Later, Swift et al. [24] tried to remedy this defect by introducing additional terms to the pressure tensor, but an analysis through the Chapman–Enskog expansion demonstrated that the lack of Galilean invariance cannot be entirely eliminated. Based on Swift et al.’s work, Inamuro et al. [25] proposed a Galilean-invariant free-energy model with the guidance of asymptotic theory. Kalarakis et al. [26] restored the Galilean invariance of the free-energy model to second-order accuracy by modifying the zero-order momentum flux tensor. Wagner and Li [27] replaced the contribution of the nonideal pressure tensor with a corrected force term and improved the Galilean invariance of the model in large velocity situations. Meanwhile, Lee and Fischer [28] reformulated the pressure form of the interaction force into a potential form and reduced the magnitude of the spurious velocity to a machine level, at the cost of including the information in next-nearest-neighbor cells. Subsequently, Guo et al. [5] spotted that the spurious velocity originates from the force imbalance at the discrete level. Based on this finding, Lou and Guo [29] applied the Lax–Wendroff scheme to the lattice Boltzmann free-energy model and successfully mitigated the effects of the force imbalance. Very recently, Guo [30] proposed a well-balanced lattice Boltzmann scheme with which the spurious velocity can be ultimately minimized to the machine accuracy. The previously mentioned improvements were carried out within the framework of the lattice Boltzmann method, which inherits its advantages such as great simplicity and high efficiency. However, the uniformity requirement on the grid types posed by the LB method prevents its application in industrial cases.

Developed in the framework of the finite volume method, the discrete unified gas-kinetic scheme (DUGKS) [31] suffers no restriction in terms of the grid types. With the information of the Knudsen number incorporated in the construction of the interface flux, the DUGKS exhibits the capability of properly modeling a wide range of fluid flows ranging from the continuum regime to the free-molecule regime [32]. Over the past decade, the DUGKS has proven its excellent performance in predicting microscale gas flows [33,34], multicomponent gas flows [35,36], turbulent flows [37,38,39], compressible flows [40,41,42], radiative heat transfer [43,44], and so forth [45]. A comparative study [46] has demonstrated the stability superiority of the DUGKS over that of the LB method in terms of nearly incompressible flows. However, the DUGKS studies centered on multiphase fluid flows remain limited [47,48] and the multiphase DUGKS has been primarily confined to the phase-field model [49]. Although Yang et al. [50] developed a pseudopotential-based DUGKS for binary fluid flow, a free parameter is needed to guarantee the isotropic property of the fluid interface. Inspired by the well-balanced LB scheme [30], Zeng et al. [51] proposed a well-balanced DUGKS for two-phase fluid flows using the free-energy model. Comparative results demonstrated the superior performance of the DUGKS over that of the LB method. Nevertheless, there is still a lack of an insightful comprehension as to the isotropic property of free-energy-based DUGKS. In this research, we elucidate the mechanism for the nonisotropic phenomena produced by the free-energy-based DUGKS using different reconstruction approaches. Then, we couple the well-balanced free-energy model with the DUGKS implemented with different reconstruction schemes to investigate practical van der Waals (vdW) fluid flows. The rest of this paper is organized as follows. In Section 2, the primitive and the well-balanced free-energy models are introduced, followed by the detailed explanation of the Strang-splitting DUGKS. The comparative numerical results, as well as brief discussions are presented in Section 3. Finally, a summary is given in Section 4.

## 2. Numerical Methodology

In this section, the first part theoretically introduces the free-energy model based on the vdW chemical potential and the second part exhaustively explains the Strang-splitting DUGKS implemented with different reconstruction schemes.

### 2.1. Free-Energy Model

Considering a multiphase system, the free-energy functional in terms of the fluid density ρ can be expressed as [14,24]

(1) $$F=\int \phi \left(\rho ,\nabla \rho \right)d\Omega_{V}=\int \left(E_{f}\left(\rho \right)+\frac{\kappa }{2}{\left|\nabla \rho \right|}^{2}\right)d\Omega_{V},$$

where $\Omega_{V}$ is the spatial region occupied by the system, ϕ(ρ,∇ρ) denotes the total free-energy density, in which $E_{f}\left(\rho \right)$ represents the bulk free-energy density, and $\frac{\kappa }{2}{\left|\nabla \rho \right|}^{2}$ signifies the interface free-energy density. The parameter κ is a positive constant determined by the interface thickness and the surface tension coefficient. Minimization of the free-energy F that is subject to the constraint of a constant mass M evolves the system towards the equilibrium condition, where

(2) $$M=\int \rho d\Omega_{v}.$$

To impose the mass constraint, a transformed free-energy functional L is constructed using the method of Lagrange multipliers:

(3) L=F−λM,

where λ is the Lagrange multiplier. Minimization of the constrained free-energy demands the corresponding first variation to be zero:

(4) δL=0,

which yields the following Euler–Lagrange equation:

(5) $$\frac{\partial \psi }{\partial \rho }-\nabla \cdot \left(\frac{\partial \psi }{\partial (\nabla \rho )}\right)=\frac{dE_{f}}{d\rho }-\kappa \nabla^{2}\rho -\lambda =0,$$

where

(6) ψ(ρ,∇ρ)=ϕ(ρ,∇ρ)−λρ.

The chemical potential $\mu_{c}$ is defined as the variation of the free-energy F with respect to the density [52]:

(7) $$\mu_{c}=\frac{\delta F}{\delta \rho }=\frac{dE_{f}}{d\rho }-\kappa \nabla^{2}\rho .$$

As the integrand of transformed free-energy L does not explicitly contain any spatial coordinates, it remains invariant regardless of the spatial translations [3]. Noether’s theorem [53] says that the invariance of the free-energy with respect to spatial translations corresponds to a conserved tensorial current J satisfying [54]:

(8) ∇·J=0,

where J is a second-rank tensor given by

(9) $$J=-\psi I+\nabla \rho ⊗\frac{\partial \psi (\rho ,\nabla \rho )}{\partial \left(\nabla \rho \right)},$$

in which I is the identity matrix. Substituting Equations (5) and (6) into Equation (9) leads to

(10) $$J=\left(\rho \mu_{c}-E_{f}-\frac{\kappa }{2}{\left|\nabla \rho \right|}^{2}\right)I+\kappa \nabla \rho \nabla \rho .$$

The bulk pressure $p_{b}$ is connected to the bulk free-energy density $E_{f}$ via the Legendre transform [54]:

(11) $$p_{b}\left(\rho \right)=\rho \frac{dE_{f}}{d\rho }-E_{f}\left(\rho \right),$$

with which the conserved current tenor J can be identified as the thermodynamic pressure tensor P in such a way that

(12) $$P\equiv J=\left(p_{b}-\kappa \rho \nabla^{2}\rho -\frac{\kappa }{2}{\left|\nabla \rho \right|}^{2}\right)I+\kappa \nabla \rho \nabla \rho .$$

With some basic algebraic manipulations, the divergence of the pressure tensor can be simplified as

(13) $$\nabla \cdot P=\rho \nabla \mu_{c}.$$

In the traditional free-energy model [28], the total effects of excess pressure accounting for the phase interactions can be represented by the following interaction force

(14) $$F=\nabla \cdot P_{0}-\nabla \cdot P=\nabla p_{0}-\rho \nabla \mu_{c},$$

where $P_{0}=p_{0}I$ denotes the pressure tensor of an ideal gas. In the well-balanced free-energy model [30], the interaction force is defined as

(15) $$F=-\rho \nabla \mu_{c}$$

in order to eliminate the force imbalance at the discrete level.

The only remaining task is to determine the bulk free-energy density $E_{f}$. In the work of Zeng et al. [51], $E_{f}$ takes a double-well form, which relates to no specific equation of state (EOS). In the current research, the bulk pressure is evaluated by the nonideal van der Waals EOS [55] expressed as

(16) $$p_{b}=\frac{\rho RT}{1-b\rho }-a\rho^{2},$$

where parameter *a* denotes the intermolecular interaction strength, parameter *b* indicates the volume correction, *R* stands for the gas constant, and *T* represents the temperature. The corresponding bulk free-energy density can be obtained by solving Equation (11):

(17) $$E_{f}\left(\rho \right)=\rho RT\ln\left(\frac{\rho }{1-b\rho }\right)-a\rho^{2}.$$

The chemical potential can then be obtained according to Equation (7):

(18) $$\mu_{c}=RT\left[\ln\left(\frac{\rho }{1-b\rho }+\frac{1}{1-b\rho }\right)\right]-2a\rho -\kappa \nabla^{2}\rho ,$$

with which the interaction force F can be evaluated. In the current research, the parameters in the vdW-EOS were set as [56] a=9/392,b=2/21,R=1. κ was fixed at 0.02 if not otherwise specified. The critical density and temperature are given as $\rho_{c}=3.5$ and $T_{c}=1/14$.

### 2.2. Strang-Splitting DUGKS

In this subsection, the evolution process of the discrete unified gas-kinetic scheme is exhaustively clarified. Then, the Strang-splitting scheme for the incorporation of the interaction force is introduced.

#### 2.2.1. Discrete Unified Gas-Kinetic Scheme

The investigation of multiphase flow problems in the current research was conducted by numerically solving the Boltzmann-BGK equation:

(19) $$\frac{\partial f}{\partial t}+\xi \cdot \nabla_{x}f=\Omega \equiv -\frac{f-f^{E}}{\tau },$$

where f=f(x,ξ,t) denotes the distribution function (DF), referring to a cluster of particles residing at position x with a velocity of ξ at time *t*, τ indicates the relaxation time, and $f^{E}$ represents the Maxwellian distribution function approached by *f* within each collision. The nondimensionalization of Equation (19) is presented in the Appendix A. The moments of distribution functions correspond to the conservative flow variables via

(20) $$\rho =\int fd\xi =\int f^{E}d\xi ,\;\rho u=\int \xi fd\xi =\int \xi f^{E}d\xi ,$$

where u denotes the velocity of the flow field. To numerically solve Equation (19), discretization of the physical and velocity space is a prerequisite. To determine the discrete velocity points along each single dimension, the three-point Gauss–Hermite quadrature is employed. The two-dimensional discrete velocity points can be derived from the tensor product of the single-dimensional velocities, which turns out to be the D2V9 velocity model commonly used in the LB community:

$$\begin{array}{cc} \xi_{i} & =\sqrt{3c_{s}^{2}}[\begin{array}{ccccccccccccccccc} 0 & \; & 1 & \; & 0 & \; & -1 & \; & 0 & \; & 1 & \; & -1 & \; & -1 & \; & 1\\ 0 & \; & 0 & \; & 1 & \; & 0 & \; & -1 & \; & 1 & \; & 1 & \; & -1 & \; & -1 \end{array}], \end{array}$$

where $\xi_{i}$ is the *i*th discrete velocity and $c_{s}=1/\sqrt{3}$ is the model speed of sound. The ideal gas pressure $p_{0}$ shown in Equation (14) relates to the density ρ through $p_{0}=\rho c_{s}^{2}$.

With the discretization of the velocity space, the Boltzmann-BGK equation turns into

(21) $$\frac{\partial f_{i}}{\partial t}+\xi_{i}\cdot \nabla_{x}f_{i}=\Omega_{i}\equiv -\frac{f_{i}-f_{i}^{E}}{\tau },$$

where the subscript *i* indicates the distribution function for particles possessing a velocity of $\xi_{i}$. Subdividing the physical space into a set of grid cells and integrating Equation (21) over a certain cell lead to

(22) $$\frac{d}{dt}\int_{V_{c}}f_{i}\left(x,t\right)dx+\int_{\partial V_{c}}\left(\xi \cdot n\right)f_{i}\left(x,t\right)dS=\int_{V_{c}}\Omega_{i}\left(x,t\right)dx,$$

where $V_{c}$ denotes the integral cell centered at position $x_{c}$, $\partial V_{c}$ denotes the surface boundary of the cell, dS is the surface element, and n is the unit vector normal to the surface element. Integrating Equation (22) over a time step of length $\Delta t=t_{n+1}-t_{n}$ yields

(23) $$f_{i}^{n+1}-f_{i}^{n}+\frac{\Delta t}{|V_{c}|}F_{i}^{n+1/2}=\frac{\Delta t}{2}\left[\Omega_{i}^{n+1}+\Omega_{i}^{n}\right],$$

where $|V_{c}|$ measures the volume of cell $V_{c}$ and $f_{i}^{n}$ and $\Omega_{i}^{n}$ approximate the cell averages of $V_{c}$ in such a way that

(24a) $$f_{i}^{n}=\frac{1}{|V_{c}|}\int_{V_{c}}f_{i}\left(x,t_{n}\right)dx,$$

(24b) $$\Omega_{i}^{n}=\frac{1}{|V_{c}|}\int_{V_{c}}\Omega_{i}\left(x,t_{n}\right)dx.$$

$F_{i}^{n+1/2}$ measures the kinetic flux at the mid-time $t_{n}+\Delta t/2$ by

(25) $$F_{i}^{n+1/2}=\int_{\partial V_{c}}\left(\xi_{i}\cdot n\right)f_{i}\left(x,t_{n}+\Delta t/2\right)dS.$$

Note that the midpoint rule is applied to compute the time integral of the kinetic flux and the trapezoidal rule is applied to evaluate the time integral of the collision term in Equation (23). To remove the implicit treatment of the collision term, two auxiliary distribution functions are introduced:

(26) $${\tilde{f}}_{i}=f_{i}-\frac{\Delta t}{2}\Omega_{i},{\tilde{f}}_{i}^{+}=f_{i}+\frac{\Delta t}{2}\Omega_{i}.$$

Substituting Equation (26) into Equation (23), we obtain a fully explicit evolution equation:

(27) $${\tilde{f}}_{i}^{n+1}={\tilde{f}}_{i}^{+,n}+\frac{\Delta t}{2}F_{i}^{n+1/2}.$$

To obtain the kinetic flux $F_{i}^{n+1/2}$, the primitive distribution function $f_{i}\left(x_{f},t_{n+1/2}\right)$ on the cell surface needs to be first evaluated. To this end, we integrate Equation (21) along the characteristic line over a time step length of δt=Δt/2:

(28) $$f_{i}\left(x_{f},t_{n+1/2}\right)-f_{i}\left(x_{f}-\xi_{i}\delta t,t_{n}\right)=\frac{\delta t}{2}\left[\Omega_{i}\left(x_{f},t_{n+1/2}\right)+\Omega_{i}\left(x_{f}-\xi_{i}\delta t,t_{n}\right)\right].$$

Note that the trapezoidal rule is once again applied for the time integral of the collision term. Similar to the treatment of Equation (23), the implicitness of Equation (28) is eliminated with the help of the following auxiliary distribution functions:

(29) $$\bar{f}=f-\frac{\delta t}{2}\Omega ,{\bar{f}}^{+}=f+\frac{\delta t}{2}\Omega .$$

Equation (28) can then be rearranged as

(30) $${\bar{f}}_{i}\left(x_{f},t_{n+1/2}\right)={\bar{f}}_{i}^{+}\left(x_{f}-\xi_{i}\delta t,t_{n}\right).$$

The auxiliary distribution function ${\bar{f}}_{i}^{+}\left(x_{f}-\xi_{i}\delta t,t_{n}\right)$ on the right-hand side of Equation (30) can be interpolated from the cell-centered ${\bar{f}}_{i}^{+}\left(x_{c},t_{n}\right)$, which could be directly constructed via Equation (29). Based on the expansion point of the Taylor series [57], the reconstruction schemes can be classified into the face-based reconstruction scheme (FRS) or the cell-based reconstruction scheme (CRS). The FRS takes the form of

(31) $${\bar{f}}_{i}^{+}\left(x_{f}-\xi_{i}\delta t,t_{n}\right)={\bar{f}}_{i}^{+}\left(x_{f},t_{n}\right)-\xi_{i}\delta t\cdot \nabla {\bar{f}}_{i}^{+}\left(x_{f},t_{n}\right),$$

in which the face-centered $f_{i}^{+}\left(x_{f},t_{n}\right)$ can be reconstructed from the cell-centered $f_{i}^{+}\left(x_{c},t_{n}\right)$ via the central difference (CD) scheme [31] or the weighted essentially non-oscillatory (WENO) scheme [58]. The upwind CRS takes the form of

(32) $$\begin{array}{c} {\bar{f}}_{i}^{+}\left(x_{f}-\xi_{i}\delta t\right)=\left\{\begin{array}{cc} {\bar{f}}_{i}^{+}\left(x_{l}\right)+\left(\delta x_{l}-\xi_{i}\delta t\right)\cdot \nabla {\bar{f}}_{i}^{+}\left(x_{l}\right)+{\left(\delta x_{l}-\xi_{i}\delta t\right)}^{2}:\nabla^{2}{\bar{f}}_{i}^{+}\left(x_{l}\right)/2,\; & \xi_{i}\cdot n\leq 0,\\ {\bar{f}}_{i}^{+}\left(x_{r}\right)+\left(\delta x_{r}-\xi_{i}\delta t\right)\cdot \nabla {\bar{f}}_{i}^{+}\left(x_{r}\right)+{\left(\delta x_{r}-\xi_{i}\delta t\right)}^{2}:\nabla^{2}{\bar{f}}_{i}^{+}\left(x_{r}\right)/2,\; & \xi_{i}\cdot n>0, \end{array}\right. \end{array}$$

where $\delta x_{l}=x_{f}-x_{l}$ measures the distance from the face center $x_{f}$ to the adjacent cell center $x_{l}$ on one side, while $\delta x_{r}=x_{f}-x_{r}$ measures the distance from the face center $x_{f}$ to the adjacent cell center $x_{r}$ on the other side. An average value is used if $\xi_{i}\cdot n=0$. After finishing the reconstruction of ${\bar{f}}_{i}^{+}\left(x_{f}-\xi_{i}\delta t,t_{n}\right)$, the face-centered auxiliary distribution function ${\bar{f}}_{i}\left(x_{f},t_{n+1/2}\right)$ can be directly obtained via Equation (30). With a straightforward transformation of Equation (29), the primitive distribution function $f_{i}\left(x_{f},t_{n+1/2}\right)$ can be calculated by

(33) $$f=\frac{2\tau }{2\tau +\delta t}\bar{f}+\frac{\delta t}{2\tau +\delta t}f^{E}.$$

The kinetic flux $F_{i}^{n+1/2}$ can then be evaluated by its definition. After that, the auxiliary distribution function ${\tilde{f}}_{i}\left(x_{c},t_{n+1}\right)$ at the next time step can be updated by Equation (27). Similarly, with a transformation of Equation (26), the primitive distribution function can be calculated by

(34) $$f=\frac{2\tau }{2\tau +\Delta t}\tilde{f}+\frac{\Delta t}{2\tau +\Delta t}f^{E}.$$

The equilibrium distribution function $f_{i}^{E}$ for the primitive free-energy model is expressed as

(35) $$f_{i}^{E}=\omega_{i}\rho \left[1+\frac{\xi_{i}\cdot u}{c_{s}^{2}}+\frac{uu:(\xi_{i}\xi_{i}-c_{s}^{2}I)}{2c_{s}^{4}}\right],$$

where $\omega_{i}=4/9$ for i=0, $\omega_{i}=1/9$ for $i=\left\{1,2,3,4\right\}$, and $\omega_{i}=1/36$ for $i=\left\{5,6,7,8\right\}$. The equilibrium distribution function $f_{i}^{E}$ for the well-balanced free-energy model is defined as

(36) $$\begin{array}{c} f_{i}^{E}=\left\{\begin{array}{cc} \rho +\omega_{0}\rho s_{0}\left(u\right),\; & i=0,\\ \omega_{i}\rho s_{i}\left(u\right),\; & i\neq 0, \end{array}\right. \end{array}$$

where

(37) $$s_{i}\left(u\right)=\left[\frac{\xi_{i}\cdot u}{c_{s}^{2}}+\frac{uu:(\xi_{i}\xi_{i}-c_{s}^{2}I)}{2c_{s}^{4}}\right].$$

Obviously, the information of macroscopic conservative variables should be first evaluated for the updating of the equilibrium distribution function. Considering the relationship between the auxiliary DF and the primitive DF presented in Equations (26) and (29), the cell-centered conservative variables are updated by

(38) $$\rho =\sum_{i}f_{i}=\sum_{i}{\tilde{f}}_{i},\;\rho u=\sum_{i}\xi_{i}f_{i}=\sum_{i}\xi_{i}{\tilde{f}}_{i},$$

and the face-centered conservative variables are updated by

(39) $$\rho =\sum_{i}f_{i}=\sum_{i}{\bar{f}}_{i},\;\rho u=\sum_{i}\xi_{i}f_{i}=\sum_{i}\xi_{i}{\bar{f}}_{i}.$$

The time step length Δt is determined by the Courant–Friedrichs–Lewy (CFL) condition:

(40) $$\Delta t=C\frac{\Delta x}{\sqrt{3c_{s}^{2}}},$$

where *C* denotes the CFL number and Δx measures the grid spacing.

#### 2.2.2. Strang-Splitting Scheme

To date, the evolution process of DUGKS without considering force terms has been exhaustively clarified. To incorporate the interaction effects between different phases, a source distribution function $f_{i}^{S}$ accounting for the force effects is introduced. To correctly recover the macroscopic hydrodynamic equation, the expression of $f_{i}^{S}$ for the primitive free-energy model is defined as

(41) $$f_{i}^{S}=\omega_{i}\left[\frac{\xi_{i}\cdot F}{c_{s}^{2}}+\frac{\mathrm{uF}:\left(\xi_{i}\xi_{i}-c_{s}^{2}I\right)}{c_{s}^{4}}\right],$$

where F is the interaction force defined in Equation (14). The expression of $f_{i}^{S}$ for the well-balanced free-energy model is defined as

(42) $$f_{i}^{S}=\omega_{i}\left[\frac{\xi_{i}\cdot F}{c_{s}^{2}}+\frac{u\left(F+c_{s}^{2}\nabla \rho \right):\left(\xi_{i}\xi_{i}-c_{s}^{2}I\right)}{c_{s}^{4}}+\frac{1}{2}\left(\frac{\xi_{i}^{2}}{c_{s}^{2}}-D\right)\left(u\cdot \nabla \rho \right)\right],$$

where D=2 is the spatial dimension. To circumvent the calculation of the interaction force on the cell interface, the Strang-splitting scheme is employed [59]. With such a treatment, the force effects are considered before and after the evolution process of the DUGKS:

(43a) $$\frac{\partial f_{i}}{\partial t}=\frac{1}{2}f_{i}^{S},$$

(43b) $$\frac{\partial f_{i}}{\partial t}+\xi_{i}\cdot \nabla_{x}f_{i}=\Omega_{i}\equiv -\frac{f_{i}-f_{i}^{E}}{\tau },$$

(43c) $$\frac{\partial f_{i}}{\partial t}=\frac{1}{2}f_{i}^{S},$$

As Equation (43b) remains identical to Equation (21), it can be solved by the DUGKS procedure addressed previously. Equations (43a) and (43c) can be numerically solved by the forward Euler method:

(44) $$f_{i}^{*}=f_{i}^{n}+\frac{\Delta t}{2}f_{i}^{S,n}.$$

The conservative variables should be accordingly updated via

(45) $$\rho^{*}=\rho^{n},u^{*}=u^{n}+\frac{\Delta t}{2}\frac{F^{n}}{\rho^{n}}.$$

The gradient operator and Laplacian operator appearing in Equations (7), (14) and (15) are implemented via the isotropic difference scheme [60].

## 3. Numerical Results

In this section, several numerical tests are conducted by the Strang-splitting DUGKS to compare the performance of the primitive free-energy model and that of the well-balanced free-energy model. The nonisotropic property caused by the reconstruction procedure in the DUGKS is especially discussed. For steady tests, the iteration terminates once the $L_{2}$-norm error satisfies

(46) $$E\left(Q\right)=\sqrt{\frac{\sum_{x}{\left|Q\left(x,t_{n}\right)-Q\left(x,t_{n-1000}\right)\right|}^{2}}{\sum_{x}{\left|Q(x,t_{n})\right|}^{2}}}<e,$$

where Q is either the flow density ρ or the flow velocity u, $t_{n-1000}$ denotes the moment 1000 time steps ahead of $t_{n}$, and *e* is the error threshold.

### 3.1. Flat Interface

**Figure 1:**
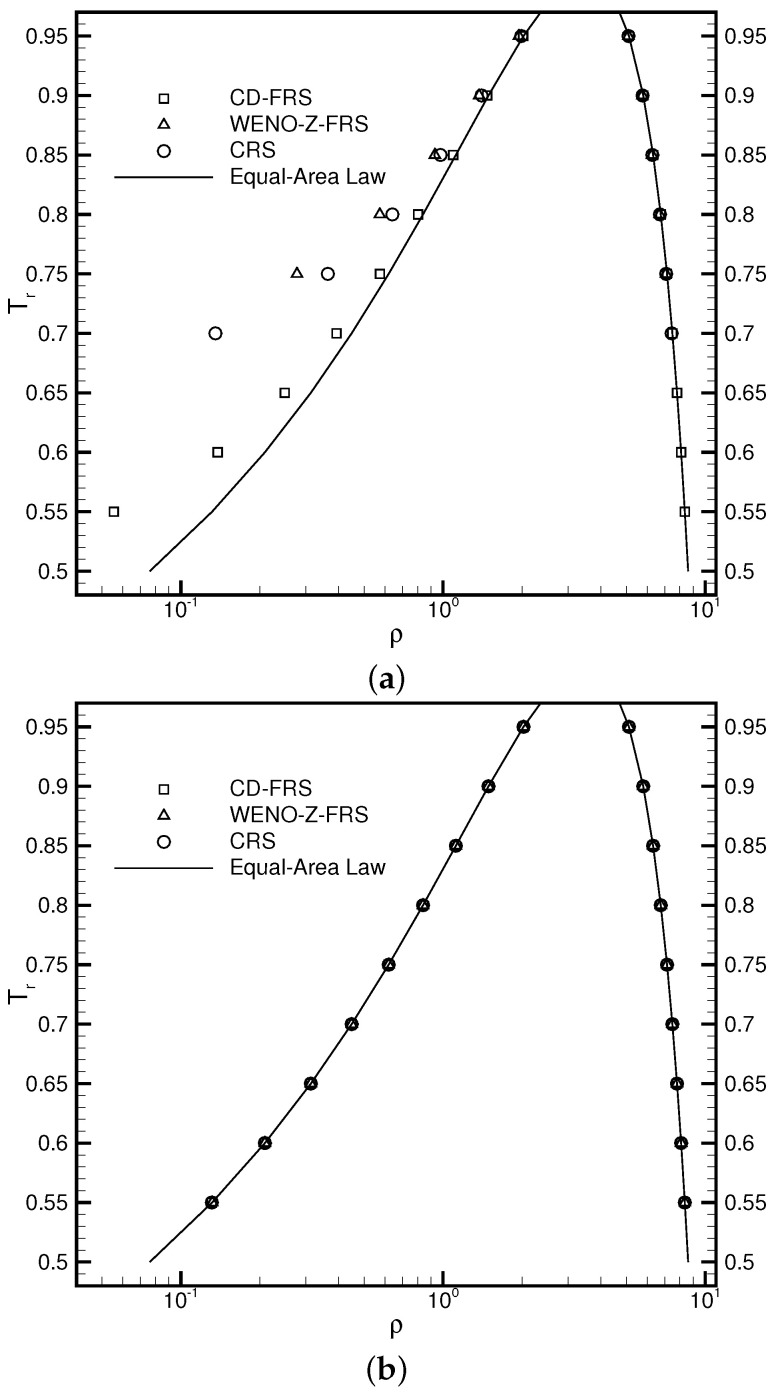
Coexisting curves produced by the DUGKS coupled with (**a**) primitive model and (**b**) well-balanced model, τ=0.3, C=0.5.

**Figure 2:**
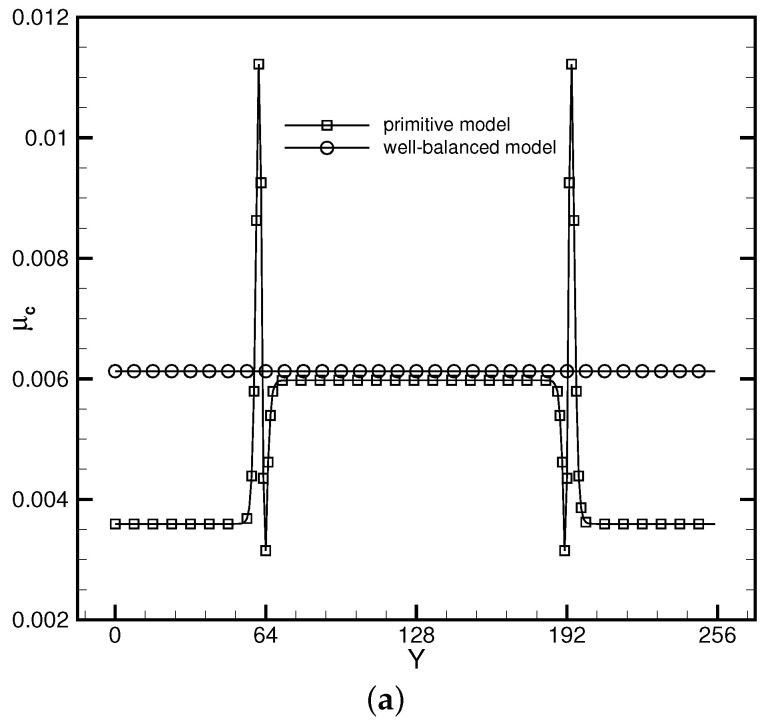

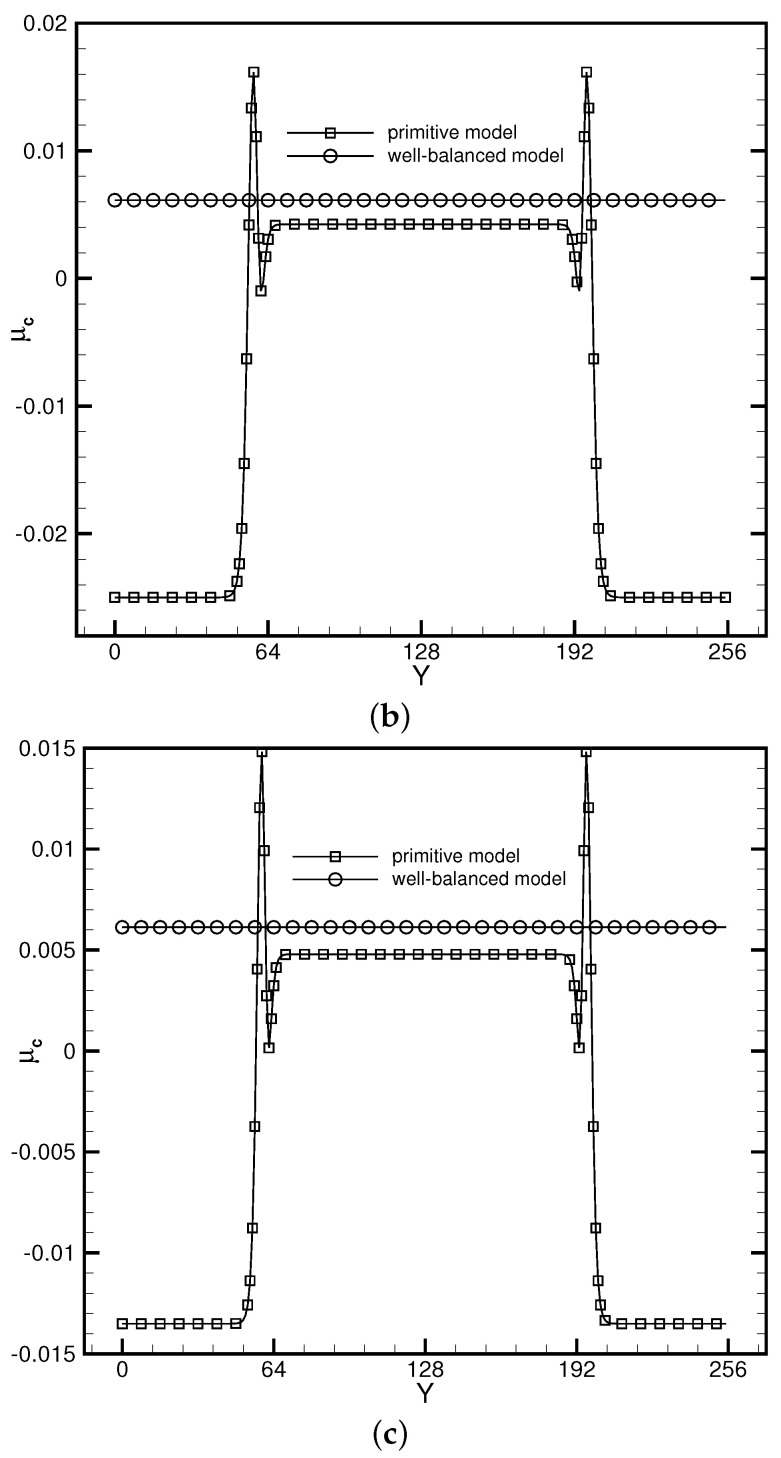
Profiles of chemical potential $\mu_{c}$ produced by the DUGKS with (**a**) CD-FRS, (**b**) WENO-Z-FRS, and (**c**) CRS, $T_{r}=0.75$, τ=0.3, C=0.5.

As a benchmark test, the flat interface has been widely applied to validate the performance of newly proposed models [30,55,56]. The computational domain is a $L_{0}\;\times \;16L_{0}$ rectangular region with $L_{0}=16$. A uniform Cartesian mesh with a grid spacing of unity is employed to subdivide this domain. Initially, the region bounded by $y_{L}=4L_{0}$ and $y_{H}=12L_{0}$ is filled up with the liquid fluid, while the rest is occupied by the gas fluid. The periodic boundary condition is applied to all the sides. The relaxation time τ was fixed at 0.3. The CFL number was set as 0.5. The reduced temperature $T_{r}=T/T_{c}$ ranged from 0.55 to 0.95. The density field is initialized by

(47) $$\rho \left(x,y\right)=\rho_{g}+\frac{\rho_{l}-\rho_{g}}{2}\left[\tanh\frac{2(y-y_{L})}{W}-\tanh\frac{2(y-y_{H})}{W}\right],$$

where *W* measures the interface thickness and $\rho_{l}$ and $\rho_{g}$ represent the liquid density and the gas density, respectively. Three reconstruction schemes were utilized to explore the influences of varying reconstruction errors on the performance of the DUGKS coupled with different free-energy models. Figure 1a illustrates the coexisting curves predicted by the DUGKS coupled with the primitive free-energy model. It can be observed that varying the reconstruction schemes offers different coexisting results. The central difference face-based reconstruction scheme (CD-FRS) provides satisfactory results in conditions of a high reduced temperature $T_{r}$. As $T_{r}$ decreases, the results deviate apparently from the theoretical results generated by the Maxwell equal-area law [61]. The WENO-Z face-based reconstruction scheme (WENO-Z-FRS) and the upwind cell-based reconstruction scheme (CRS) produce inconsistent results in conditions of high $T_{r}$. As $T_{r}$ decreases, both of them suffer from the stability problem. The fact that different reconstruction schemes generate divergent outcomes results from the force imbalance in the primitive free-energy model [30]. As the standard LB method involves no reconstruction process, the influences of the force imbalance on the numerical results remain limited. When it is coupled with numerical methods containing a reconstruction process, the effect of the force imbalance becomes amplified by the reconstruction errors. Figure 1b illustrates the results produced by the DUGKS coupled with the well-balanced free-energy model, in which the force imbalance was entirely eliminated. It can be identified that the coexisting densities predicted by different reconstruction schemes coincide exactly with the theoretical results. Moreover, the DUGKS implemented with different reconstruction schemes performs equally well in conditions of a low reduced temperature $T_{r}$, which demonstrates the fundamental accuracy and stability of this method. Figure 2 illustrates the comparative chemical potential profiles produced by the DUGKS coupled with different free-energy models at $T_{r}=0.75$, τ=0.3, C=0.5. Regardless of the reconstruction schemes utilized, the well-balanced free-energy-based DUGKS provides a nearly constant chemical potential profile, while the primitive free-energy-based DUGKS offers a varied chemical potential profile across the interfaces. Taking a closer look at the comparative profiles, we can identify that the chemical potential value produced by the DUGKS coupled with the primitive model varies along with the reconstruction schemes used, which should be attributed to the differences in the reconstruction errors. The chemical potential produced by the DUGKS coupled with the well-balanced model holds a nearly constant value of 0.006126, which demonstrates the excellent performance of the well-balanced DUGKS in predicting steady two-phase systems governed by free-energy theory.

### 3.2. Quiescent Droplet

**Figure 3:**
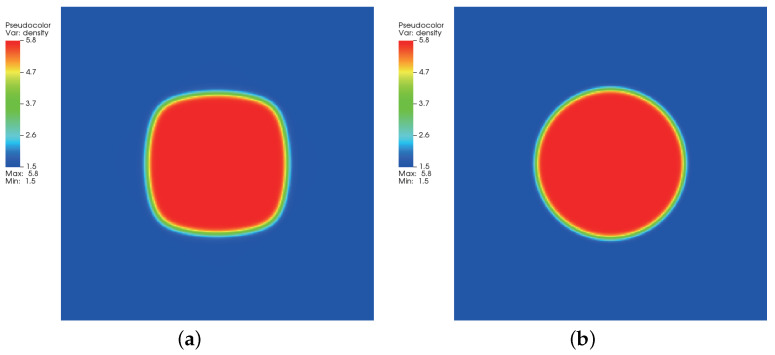
Density contours produced by DUGKS implemented with CD-FRS coupled with (**a**) primitive model and (**b**) well-balanced model, $T_{r}=0.9$, τ=0.6, C=0.5.

**Figure 4:**
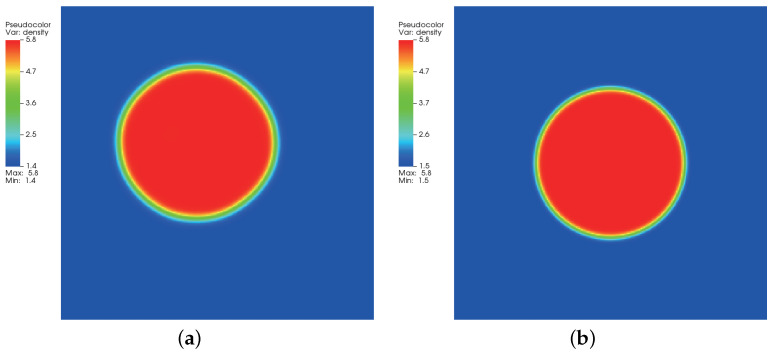
Density contours produced by DUGKS implemented with WENO-Z-FRS coupled with (**a**) primitive model and (**b**) well-balanced model, $T_{r}=0.9$, τ=0.6, C=0.5.

**Figure 5:**
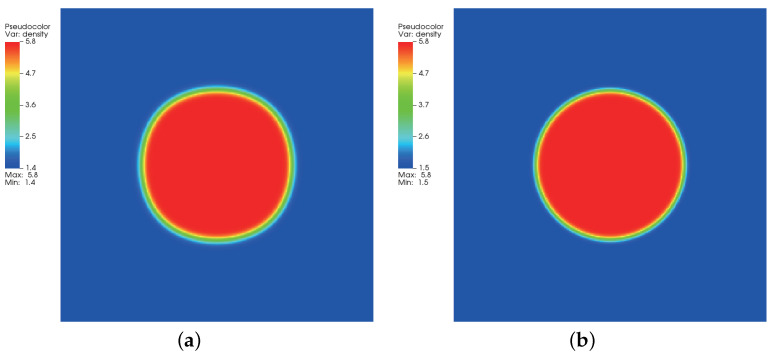
Density contours produced by DUGKS implemented with CRS coupled with (**a**) primitive model and (**b**) well-balanced model, $T_{r}=0.9$, τ=0.6, C=0.5.

**Figure 6:**
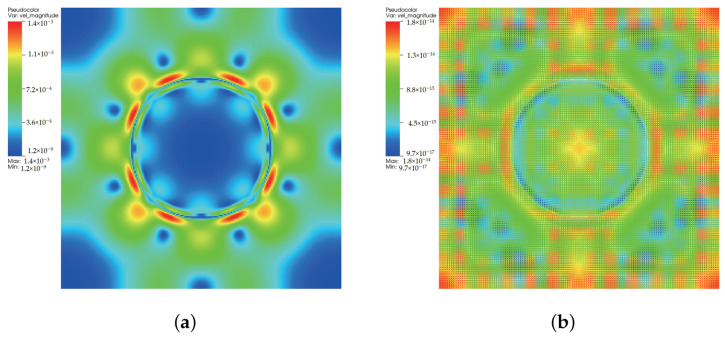
Velocity contours produced by DUGKS implemented with CRS coupled with (**a**) primitive model and (**b**) well-balanced model, $T_{r}=0.9$, τ=0.6, C=0.5.

The quiescent droplet test serves as one of the fundamental benchmarks for validating the basic capability of the newly proposed multiphase methods. A circular droplet is initially placed at the center of an $L_{0}\;\times \;L_{0}$ square domain, with $L_{0}=256$. A uniform Cartesian mesh is used to discretize the physical domain, with the grid spacing Δx fixed at unity. The density field is initialized according to

(48) $$\rho \left(x,y\right)=\frac{\rho_{l}+\rho_{g}}{2}-\frac{\rho_{l}-\rho_{g}}{2}\tanh\left[\frac{2\left(\sqrt{|x-x_{c}{|}^{2}+{\left|y-y_{c}\right|}^{2}}-R_{d}\right)}{W}\right],$$

where $\rho_{l}$ and $\rho_{g}$ correspond, respectively, to the coexisting liquid and gas densities, $\left(x_{c},y_{c}\right)$ indicates the center location of the square domain, $R_{d}$ denotes the droplet radius, and *W* measures the interface thickness. The computing process terminates once the $L_{2}$-norm error of density evaluated by Equation (46) is below $10^{-10}$. Figure 3, Figure 4 and Figure 5 illustrate the density contours produced by the DUGKS coupled with different free-energy models and implemented by various reconstruction schemes at $T_{r}=0.9,\tau =0.6,C=0.5$. The interfaces produced with the primitive free-energy model suffer from the nonisotropic problem regardless of the reconstruction scheme utilized, which is caused by the force imbalance addressed previously. The second-order central-difference face-based reconstruction scheme (CD-FRS) evolves the initially circular interface into a roughly square interface, which should be attributed to the relatively large reconstruction errors. With a long time evolution, the fifth-order WENO-Z face-based reconstruction scheme (WENO-Z-FRS) shifts the quiescent droplet away from the center position. The interface profile deforms less than that produced by the CD-FRS, which might be attributed to the low level of reconstruction errors of WENO-Z. The interface profile generated by the third-order cell-based reconstruction scheme (CRS) is rather close to circular, which is due to the less nonisotropic reconstruction errors. A similar phenomenon can be observed in the results produced by the pseudopotential-based DUGKS. The interface profiles produced with the well-balanced free-energy model preserve a universal isotropic property across all reconstruction schemes, which demonstrates the elimination of the force imbalance. Figure 6 illustrates the contour of the velocity field produced by the DUGKS implemented with the CRS at $T_{r}=0.9,\tau =0.6,C=0.5$. When the steady-state is reached, the velocity field produced by the primitive model exhibits a typical patten of large spurious currents, while the velocity field obtained with the well-balanced model provides spurious currents of machine accuracy. The excellent performance of the well-balanced DUGKS is thus verified by the comparative results.

**Figure 7:**
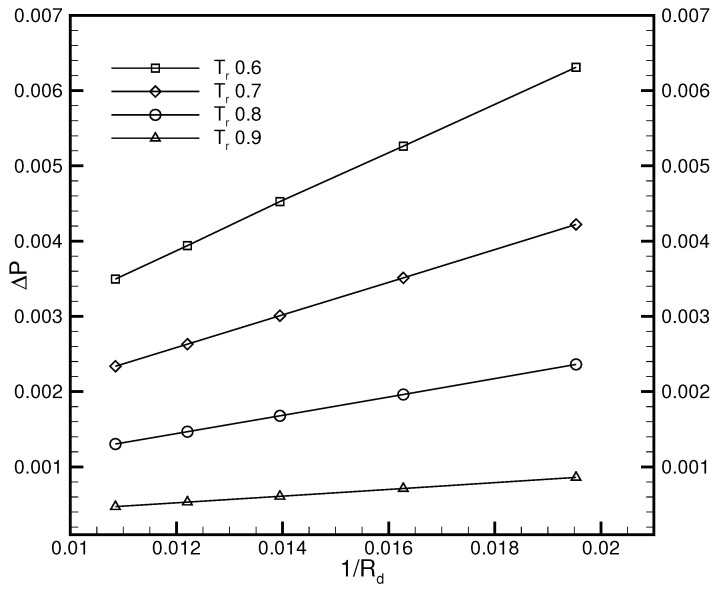
Validation of Laplace’s law, τ=0.3, C=0.8.

To quantitatively assess its capability, Laplace’s law is validated by the well-balanced DUGKS implemented with the CRS. Figure 7 illustrates the relations between the pressure jump ΔP and the reciprocal of radius $R_{d}$ obtained at τ=0.3, C=0.8. The linear relation can be clearly identified from the results, which conforms to Laplace’s law: $\Delta P=\sigma /R_{d}$. The chemical potential varies along with the reduced temperature $T_{r}$, which results in the alteration of the surface tension coefficient σ. The CFL number was set as 0.8, at which the FRS fails to operate properly. The stability superiority of the CRS over that of the FRS in the condition of a large time step size makes it more appealing for multiphase flow simulations.

### 3.3. Spinodal Decomposition

**Figure 8:**
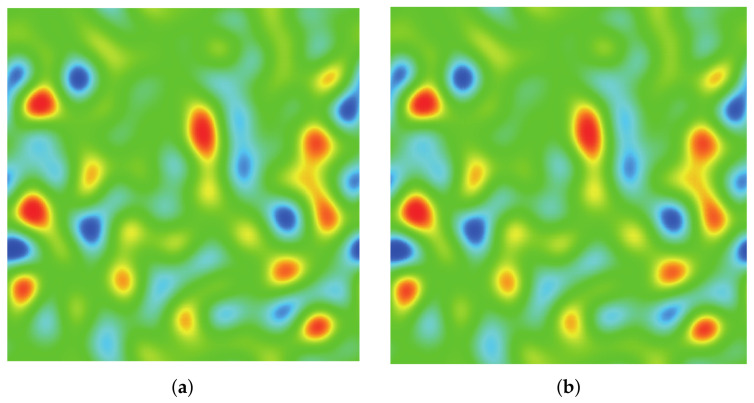
Snapshots of the density distribution produced by the DUGKS implemented with (**a**) CD-FRS and (**b**) CRS, $T_{r}=0.9$9 τ=0.6, C=0.5, *t* = 2500.

**Figure 9:**
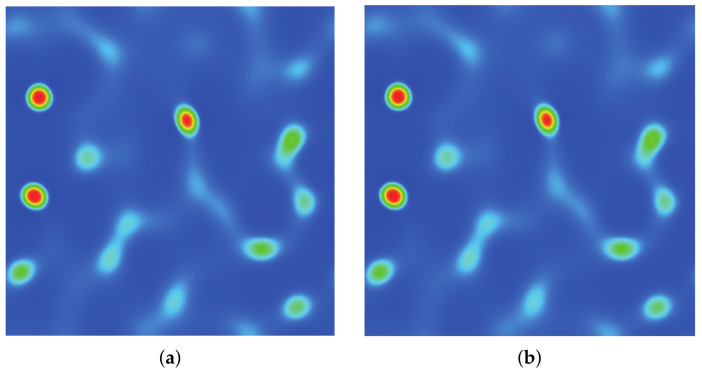
Snapshots of the density distribution produced by the DUGKS implemented with (**a**) CD-FRS and (**b**) CRS, $T_{r}=0.9$, τ=0.6, C=0.5, *t* = 6000.

**Figure 10:**
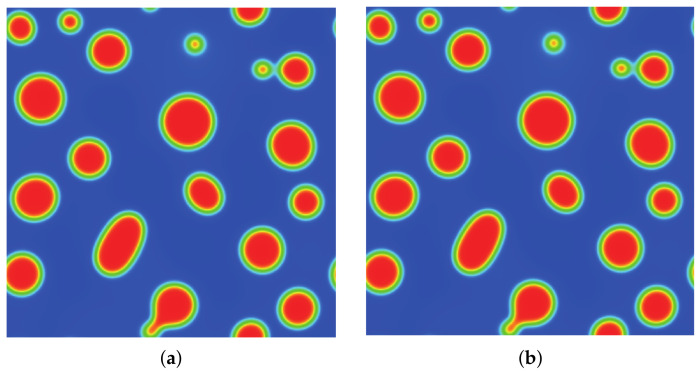
Snapshots of the density distribution produced by the DUGKS implemented with (**a**) CD-FRS and (**b**) CRS, $T_{r}=0.9$, τ=0.6, C=0.5, *t* = 7500.

**Figure 11:**
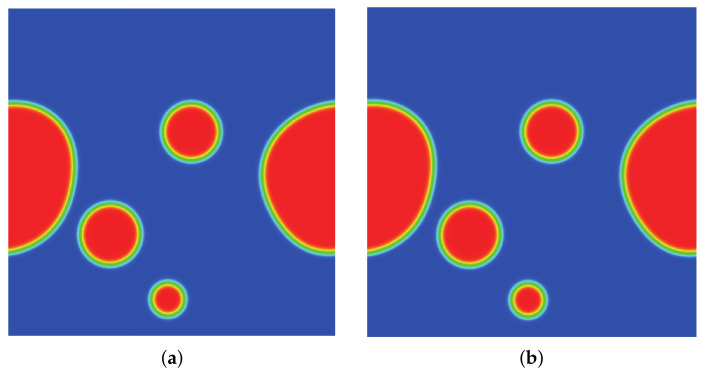
Snapshots of the density distribution produced by the DUGKS implemented with (**a**) CD-FRS and (**b**) CRS, $T_{r}=0.9$, τ=0.6, C=0.5, *t* = 25,000.

**Figure 12:**
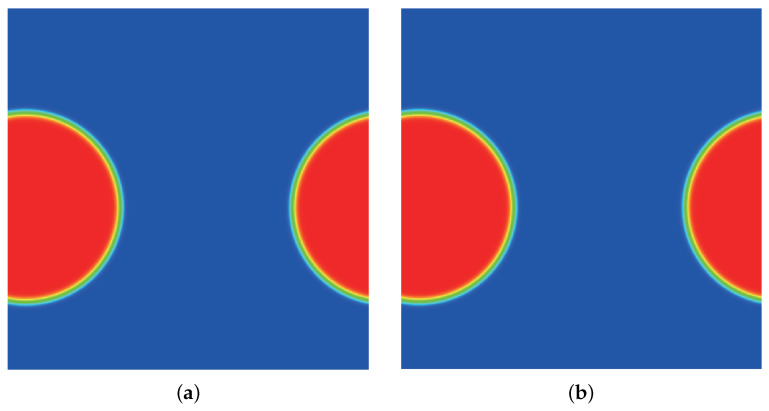
Snapshots of the density distribution produced by the DUGKS implemented with (**a**) CD-FRS and (**b**) CRS, $T_{r}=0.9$, τ=0.6, C=0.5, t= 250,000.

Previous benchmark tests were limited to steady-state problems. Here, the spinodal decomposition test was adopted to assess the capability of DUGKS in dealing with transient problems. The computational domain is an $L_{0}\;\times \;L_{0}$ square region subdivided by the uniform Cartesian mesh. The grid spacing Δx=1, and the characteristic length $L_{0}=512$. The periodic boundary condition was applied to all the sides. The density field is initialized by

(49) $$\rho \left(x,y\right)=\left(\rho_{l}+\rho_{g}\right)/3+\mathrm{random}\left(0,1\right)/100,$$

where $\rho_{l}$ and $\rho_{g}$ represent the liquid density and the gas density and random(0,1) creates density fluctuations that induce the spinodal decomposition process. Figure 8, Figure 9, Figure 10, Figure 11 and Figure 12 illustrate the snapshots of the density distribution produced by the DUGKS coupled with the well-balanced free-energy model at $T_{r}=0.9$, τ=0.6, C=0.5. In the early stages, the tiny fluctuations generate local inhomogeneities, which initialize the phase separation. As the system evolves, the inhomogeneities drive the material of the heavy fluid into small droplets and interfaces separating different phases begin to emerge. With the continual evolution of the whole system, some of these droplets gradually coalesce into large ones. Eventually, a complete quiescent droplet is formed. It can be identified that the results produced by the central difference face-based reconstruction scheme (CD-FRS) are nearly identical to those generated by the third-order cell-based reconstruction scheme (CRS), which demonstrates the consistent behaviors of the well-balanced DUGKS. The WENO-Z face-based reconstruction scheme (WENO-Z-FRS) fails to provide a converged solution in such a condition. Moreover, the well-balanced DUGKS fails to predict the evolution process of the spinodal decomposition when the reduced temperature is below 0.8. To investigate the multiphase flow dynamics by the well-balanced DUGKS, further improvements are required.

### 3.4. Droplet Coalescence

**Figure 13:**
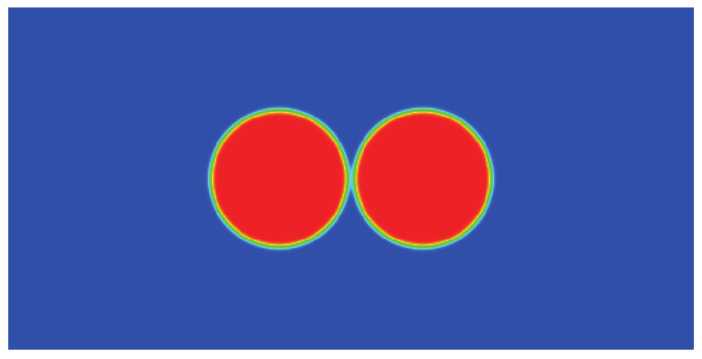
Initial distribution of the density field.

**Figure 14:**
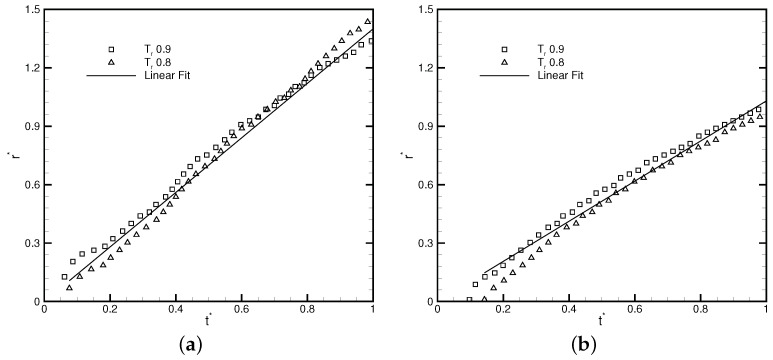
Radius variation of the liquid bridge with regard to the dimensionless time produced by DUGKS coupled with (**a**) primitive model and (**b**) well-balanced model, τ=0.3, C=0.8.

**Figure 15:**
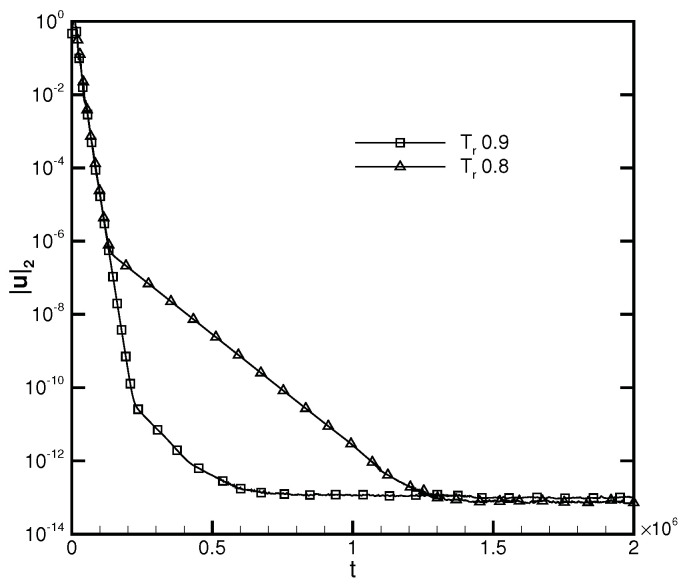
$L_{2}$-norm of the velocity field produced by the well-balanced DUGKS with the evolution of time, τ=0.3, C=0.8.

**Figure 16:**
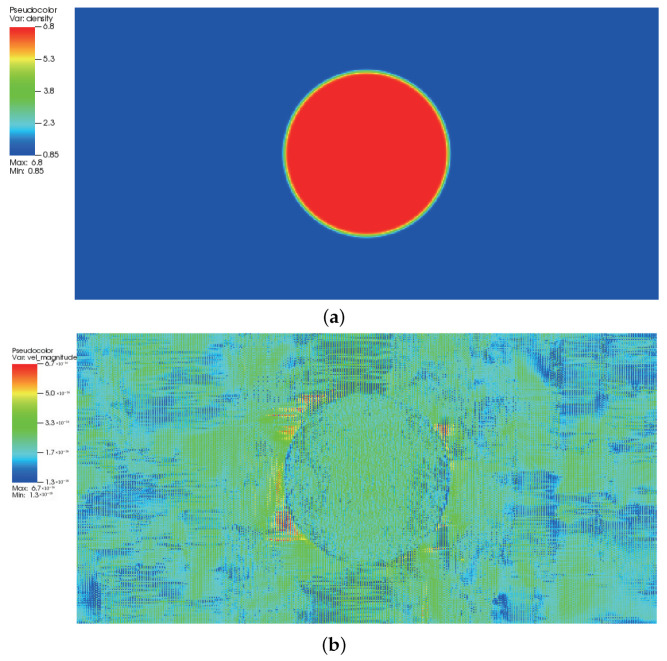
Contours of (**a**) density field and (**b**) velocity field produced by the well-balanced DUGKS at $t=6\;\times \;10^{6}$, $T_{r}=0.8$, τ=0.3, C=0.8.

Simulations of the droplet coalescence phenomenon were used to further investigate the capacity of the well-balanced DUGKS for transient problems. The computational domain is a rectangle $2L_{0}\;\times \;L_{0}$ domain with $L_{0}=256$. The domain was subdivided into finite grid cells by a uniform Cartesian mesh with a grid spacing of unity. To avoid wall boundary influence, a periodic boundary condition was used in all directions. Initially, two circular droplets were arranged in accordance with [51]

(50) $$\rho \left(x,y\right)=\frac{\rho_{l}+\rho_{g}}{2}+\frac{\rho_{l}-\rho_{g}}{2}\left[1-\tanh\left(\frac{2d_{A}}{W}\right)-\tanh\left(\frac{2d_{B}}{W}\right)\right],$$

where $\rho_{l}$ and $\rho_{g}$ correspond separately to the liquid and gas densities, *W* measures the interface thickness, and $d_{A}$ and $d_{B}$ are defined as

(51) $$d_{A}=\sqrt{{\left(x-x_{A}\right)}^{2}+{\left(y-y_{A}\right)}^{2}}-R_{0},\;d_{B}=\sqrt{{\left(x-x_{B}\right)}^{2}+{\left(y-y_{B}\right)}^{2}}-R_{0},$$

in which $R_{0}$ denotes the droplet radius and $\left(x_{A},y_{A}\right)=\left(L_{0}-R_{0}-W/2,L_{0}/2\right)$ and $\left(x_{B},y_{B}\right)=\left(L_{0}+R_{0}+W/2,L_{0}/2\right)$ represent the central position of droplets A and B, respectively. Other parameters were set as κ=0.02, $R_{0}=0.2L_{0}$, W=5, and τ=0.3. The initial profile of two droplets is illustrated in Figure 13. The coalescence process starts when the droplets come in contact with each other. As the process continues, a liquid bridge of radius $r_{b}$ that connects the two droplets is formed [51]. Previous research [62] identified the linear relation between the scaled radius $r^{*}$ and the dimensionless time $t^{*}$, with

(52) $$r^{*}=r_{b}/R_{0},\;t^{*}=t/\sqrt{\rho_{l}R_{0}^{3}\sigma },$$

where σ is the surface tension coefficient. According to the validation of Laplace’s law illustrated in Figure 7, the surface tension coefficient is 0.1203 for $T_{r}=0.8$ and 0.0435 for $T_{r}=0.9$. Figure 14 presents the radius variation of the liquid bridge with regard to the dimensionless time $t^{*}$. The linear coefficient for the fitting result provided by the DUGKS using the primitive model is 1.4, while the linear coefficient for the fitting result produced with the well-balanced model is 1.03, which is in good agreement with the result predicted by Zeng et al. [51]. The evolution of the $L_{2}$-norm of the velocity field produced by the DUGKS using the well-balanced model at $T_{r}=0.8$ and $T_{r}=0.9$ is shown in Figure 15. It can be identified that the $L_{2}$-norm of the velocity field reaches a magnitude of $10^{-14}$, which is consistent with the results predicted at the steady-state. Figure 16 illustrates the density and velocity contours produced by the well-balanced DUGKS at $t=6\;\times \;10^{6}$, $T_{r}=0.8$, τ=0.3, C=0.8. It can be observed that the interface maintains excellent isotropy and the velocity field holds a maximum magnitude of $10^{-16}$, which demonstrates the excellent ability of the well-balanced DUGKS. However, it is important to note that when the lowered temperature $T_{r}$ is less than 0.7, the DUGKS is unable to predict the coalescence process. More efforts are required to increase the stability of the well-balanced DUGKS.

## 4. Conclusions

A free-energy-based discrete unified gas-kinetic scheme (DUGKS) was developed by coupling the well-balanced free-energy model with the DUGKS to investigate the van der Waals fluid. The performance of this well-balanced scheme was compared against the counterpart of the DUGKS coupled with the primitive free-energy model. Comparative results produced with different reconstruction schemes demonstrated the force imbalance in the primitive free-energy model, which prevents its direct application to the DUGKS. By coupling the well-balanced free-energy model with the DUGKS, the amplified effects of the force imbalance are entirely eliminated and the influences of nonisotropic reconstruction errors on the fluid interfaces are totally removed. Numerical tests of a flat interface, quiescent droplet, spinodal decomposition, and droplet coalescence were adopted to assess the performance of the DUGKS coupled with the well-balanced free-energy model. Coexisting density curves and Laplace’s law were utilized to evaluate its capability quantitatively. It was proven that the well-balanced DUGKS could always produce consistent results despite the reconstruction schemes utilized in steady cases. When dealing with transient problems, the reconstruction scheme employing WENO-Z to evaluate face unknowns tends to be more unstable. When the reduced temperature is below 0.7, the DUGKS coupled with the well-balanced free-energy model suffers from stability problems. Further improvements are required to apply this scheme to predict transient multiphase fluid flows.

## Data Availability

The data that support the findings of this study are available from the corresponding author upon reasonable request.

## Appendix A. Nondimensionalization of the Boltzmann-BGK Equation

The nondimensionalization process of the Boltzmann-BGK equation is analyzed in this part. The dimensional Boltzmann-BGK equation with a source term reads

(A1) $$\frac{\partial f}{\partial t}+\xi \cdot \nabla_{x}f=-\frac{1}{\tau }\left(f-f^{E}\right)+f^{S},$$

where *f* represents the distribution function, *t* is the time, x is the position, ξ is the particle velocity, τ is the relaxation time, $f^{E}$ indicates the equilibrium distribution function, and $f^{S}$ accounts for the source distribution function. Introducing the characteristic length $l_{c}$, the characteristic velocity $u_{c}$, the characteristic density $\rho_{c}$, and multiplying Equation (A1) by $l_{c}/\left(\rho_{c}u_{c}\right)$ on both sides, we have

(A2) $$\frac{\partial f^{*}}{\partial t^{*}}+\xi^{*}\cdot \nabla_{x^{*}}f^{*}=-\frac{l_{c}}{u_{c}\tau }\left(f^{*}-f^{E,*}\right)+f^{S,*},$$

where

(A3) $$t^{*}=\frac{tu_{c}}{l_{c}},\xi^{*}=\frac{\xi }{u_{c}},x^{*}=\frac{x}{l_{c}},f^{*}=\frac{f}{\rho_{c}},f^{E,*}=\frac{f^{E}}{\rho_{c}},f^{S,*}=\frac{f^{S}l_{c}}{\rho_{c}u_{c}}.$$

As the relaxation time τ is evaluated by $\tau =\mu /\rho_{c}c_{s}^{2}$, where μ is the dynamic viscosity, the multiplier $l_{c}/\left(u_{c}\tau \right)$ becomes

(A4) lcucτ=lcρccs2ucμ=ReMa,

where

(A5) Ma=uccs,Re=ρclccsμ.

Equation (A2) turns into

(A6) ∂f∗∂t∗+ξ∗·∇x∗f∗=−ReMaf∗−fE,∗+fS,∗,

which is the dimensionless Boltzmann-BGK equation. In multiphase simulation involving droplet dynamics, the Reynolds number is generally defined as

(A7) Red=ρcuclcμ.

With this definition, the dimensionless Boltzmann-BGK equation becomes

(A8) ∂f∗∂t∗+ξ∗·∇x∗f∗=−RedMa2f∗−fE,∗+fS,∗.
